# Prevalence of anxiety, depressive and insomnia symptoms among the different groups of people during COVID-19 pandemic: An overview of systematic reviews and meta-analyses

**DOI:** 10.3389/fpsyg.2022.1024668

**Published:** 2022-11-16

**Authors:** Qi Zou, Yuning Tang, Cheng Jiang, Pengyang Lin, Jinhui Tian, Shougang Sun

**Affiliations:** ^1^Department of Cardiology, Lanzhou University Second Hospital, Lanzhou, China; ^2^Evidence-Based Medicine Center of Lanzhou University, Lanzhou, China

**Keywords:** COVID-19, anxiety, depression, insomnia, systematic review, AMSTAR 2

## Abstract

Since the pandemic of the novel 2019 coronavirus disease (COVID-19), in addition to the harm caused by the disease itself, the psychological damage caused to the public by the pandemic is also a serious problem. The aim of our study was to summarize the systematic reviews/meta-analyses (SRs/MAs) of the prevalence of anxiety, depression and insomnia in different populations during the COVID-19 pandemic and to qualitatively evaluate these SRs/MAs. We searched the Cochrane Library, PubMed and Web of Science to obtain SRs/MAs related to anxiety, depression, and insomnia in different populations during the COVID-19 pandemic. The main populations we studied were healthcare workers (HCWs), college students (CSs), COVID-19 patients (CPs), and the general populations (GPs). A subgroup analysis was performed of the prevalence of psychological disorders. A total of 42 SRs/MAs (8,200,330 participants) were included in calculating and assessing the prevalence of anxiety, depression, and insomnia in these populations. The results of subgroup analysis showed that the prevalence of anxiety in different populations were: HCWs (20–44%), CSs (24–41%), CPs (15–47%), and GPs (22–38%). The prevalence of depression were: HCWs (22–38%), CSs (22–52%), CPs (38–45%), and GPs (16–35%), statistically significant differences between subgroups (*p* < 0.05). The prevalence of insomnia were: HCWs (28–45%), CSs (27–33%), CPs (34–48%), and GPs (28–35%), statistically significant differences between subgroups (*p* < 0.05). The comparison revealed a higher prevalence of psychological disorders in the CP group, with insomnia being the most pronounced. The methodological quality of the included SRs/MAs was then evaluated using AMSTAR 2 tool. The results of the methodological quality evaluation showed that 13 SRs/MAs were rated “medium,” 13 were rated “low,” and 16 were rated “very low.” Through the subgroup analysis and evaluation of methodological quality, we found a higher prevalence of insomnia than anxiety and depression among the psychological disorders occurring in different populations during the pandemic, but the sample size on insomnia is small and more high-quality studies are needed to complement our findings.

## Introduction

Since the outbreak of the COVID-19 pandemic in December 2019, the suddenness of the COVID-19 pandemic and the lack of effective preventive measures at the beginning has led to the rapid spread of the pandemic worldwide, and by the end of Jun 2022, the COVID-19 pandemic outbreak has caused about 50 million infections and 6 million deaths in more than 200 countries worldwide, resulting in incalculable human casualties and economic losses. As a result, the enormous toll of the COVID-19 pandemic has led to a significant increase in the incidence of psychological disorders in different segments of society, the most common of which are anxiety, depression, and insomnia (Bao et al., 2020). Psychological disorders occur mainly in healthcare workers (HCWs), college students (CSs), COVID-19 patients (CPs), and the general populations (GPs), who are also the groups more severely affected by the pandemic (Alsubaie et al., 2019; Chen Q. et al., 2020; Pfefferbaum and North, 2020; Wang C. et al., 2020; Zhang et al., 2020).

The fact that the CPs are already infected themselves, coupled with the increasing number of deaths each day, has led to anxiety for their lives and panic, coupled with being in quarantine and isolated from the outside world, creating a severe sense of isolation (Alsubaie et al., 2019). The large number of casualties caused by the pandemic has increased the burden and psychological stress on HCWs. In addition, many HCWs have unfortunately also been infected due to prolonged close contact with the CPs (Wang J. et al., 2020). It has been reported that more than 17,000 HCWs have died from COVID-19 (Huang et al., 2020; Wu et al., 2020). The prolonged pandemic has also caused serious psychological disorders among CSs. The lack of knowledge about the pandemic and excessive attention to internet information has increased anxiety and depression among some students, in addition to the lack of physical exercise and long hours of screen study, with senior students worrying about their graduation (Han et al., 2020). Since most factories and companies and other related places cannot function normally during the pandemic, a large number of people are isolated from home of economic resources and lack normal social communication, they are prone to psychological disorders. In addition, the shortage of food with high prices caused severe anxiety and depression in the minds of the people (Kawohl and Nordt, 2020; Lone and Ahmad, 2020; Browning et al., 2021).

For the above, there have been many systematic reviews/meta-analyses (SRs/MAs) incorporating different cross-sectional studies to calculate the prevalence of some common psychological disorders in different populations during the pandemic, especially the pooled prevalence of anxiety, depression, and insomnia. There are many SRs/MAs on the majority of anxiety, depression, and insomnia in different populations during the COVID-19 pandemic, but these reviews differ in quality and design. Therefore, it is needed to assess the methodological quality of these SRs/MAs, summarize the evidence for the important outcomes included in the SRs/MAs, state the conclusions of these SRs/MAs and combine these results to produce more accurate data for a large sample size.

## Materials and methods

### Sources of literature and search strategy

**FIGURE 1:**
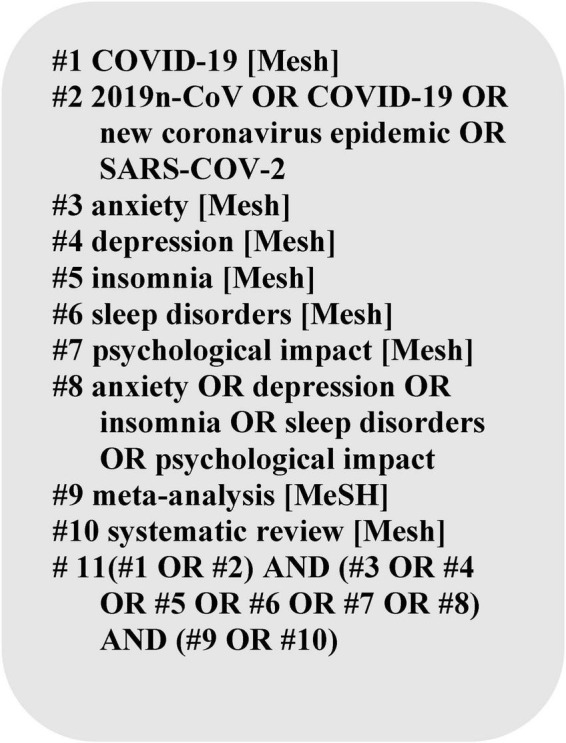
Search strategies.

To identify the included literature, we searched for SRs/MAs published in the Cochrane Library, PubMed and Web of Science from December 2019 to June 2022 related to our study topic, without applying any language restrictions and the search terms included all identified keywords (“2019n-CoV,” “new coronavirus pandemic,” “COVID-19,” “anxiety,” “depression,” “insomnia,” “sleep disorders,” “psychological impact”) and adjusted for each database. The detailed search strategy is shown in Figure 1.

### Inclusion and exclusion criteria

Systematic reviews/MAs were included if they met the following criteria: (1) published since the outbreak of COVID-19 in December 2019. (2) the studies included in the report involved study populations that experienced the COVID-19 pandemic. (3) the determination of anxiety, depression, and insomnia in the study populations included in the report was subject to the use of an authoritative assessment tool for psychological disorders. (4) the prevalence of anxiety, depression, or insomnia in the study population was provided. (5) the type of studies included in the report were cross-sectional studies. (6) The type of publication of the literature is systematic reviews/meta-analyses. We excluded literature reviews that (1) used informal and subjective methods to collect and interpret evidence, reviews, and non-peer reviews; and (2) our study population did not include pregnant mothers, chronic patients, elderly and children, so these populations were excluded from the study.

### Study selection

All the retrieved literature was imported into NoteExpress software to identify and remove duplicate studies. Then the titles and abstracts of the papers were browsed to initially exclude literature that was far from the purpose of our research. Two evaluators (QZ and YT) then independently read and evaluated the full articles. Those that did not meet the pre-determined inclusion criteria were excluded, with a third evaluator (SS) making the determination when no agreement could be reached on any of them.

### Data extraction

Two evaluators (CJ and PL) independently extracted data from the included reports based on a pre-designed Excel spreadsheet for quality assessment and data analysis. The data extraction spreadsheet summarized key characteristics: (1) year of publication and authors; (2) number of included studies; (3) total sample size; (4) outcome indicators and prevalence; (5) quality assessment tools; and (6) journal of publication. When agreement could not be reached on data from the literature, a third evaluator (SS) made the determination. When necessary, additional information was obtained from the original cross-sectional study reports.

### Methodological quality assessment

The methodological quality of the included studies was independently evaluated by two evaluators using the AMSTAR 2 tool (Shea et al., 2017). The tool contains 16 entries, of which entries 2, 4, 7, 9, 11, 13, and 15 are vital entries, and the results were classified into 3 levels “satisfied,” “partially satisfied,” and “not satisfied.” AMSTAR 2 tools of satisfied and partially satisfied ≥70% were considered to be more complete for entry reporting. AMSTAR 2 Scoring Quality Levels. See Supplementary Table 1 for definitions of quality levels.

### Managing overlapping systematic reviews

Some SRs/MAs may have repeatedly included the same study when discussing the same or similar topics. Multiple inclusion of the same study can lead to biased outcome data. Therefore, when conducting evidence summaries, these repeatedly included studies can have a greater impact on our primary outcomes. According to Cochrane’s guidance (Higgins et al., 2020), results from all relevant studies should be included if the purpose of the overview is to present and describe the current body of evidence on a topic, so we did not exclude overlapping systematic reviews. However, to avoid greater bias in the final results, we presented the results of the included SRs/MAs using only forest plots and did not pool the results.

### Statistical analysis

To investigate the prevalence of anxiety, depression, and insomnia worldwide during the COVID-19 pandemic, we divided the target population into four subgroups (HCWs, CSs, CPs, GPs) and analyzed the prevalence of anxiety, depression, and insomnia in each subgroup. Meta-analysis was performed using Revman 5.4 software, with prevalence and its 95% *CI* as statistical effect measures. Heterogeneity among the included studies was analyzed using the χ^2^ test (test level α = 0.1), while the magnitude of heterogeneity was quantified by combining *I*^2^. When *I*^2^ > 50% or *p* < 0.10, a random-effects model was used, otherwise a fixed-effects model was used. To evaluate the robustness of the results of group comparisons, we performed statistical tests on the results of group comparisons, suggesting statistically significant differences when the *p* < 0.05. Microsoft Excel 2018 was used to record the relevant data and bubble plot of the results of the methodological quality assessment of the literature. We used percentages to describe the prevalence of psychological disorders.

## Results

### Study identification

**FIGURE 2:**
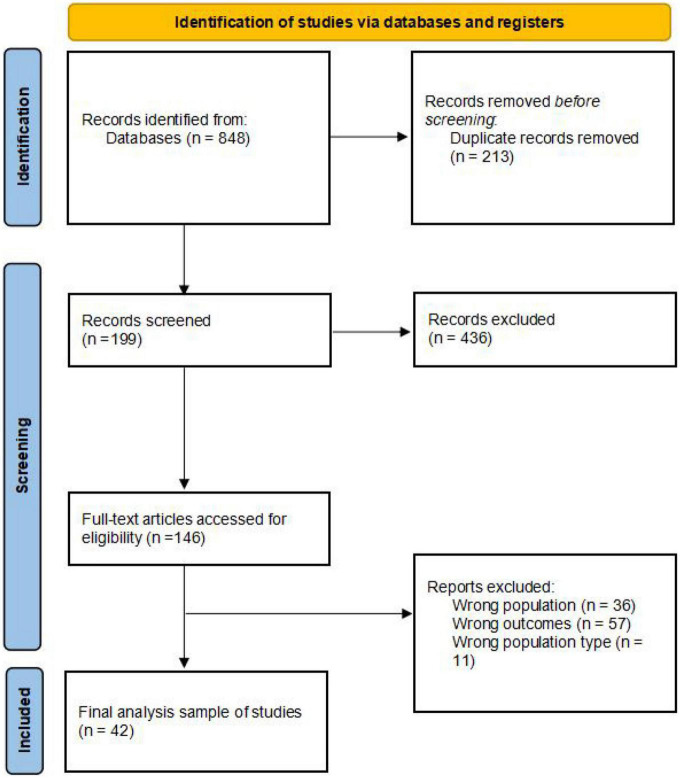
The flow chart of the trial selection.

A total of 848 records were identified with our search. Of these, 635 were screened after the removal of duplicates. After screening titles and abstracts, 436 records were excluded. The full text of the remaining 199 records was retrieved for further scrutiny. Of these, 157 were excluded because they did not fulfill the eligibility criteria. Finally, 42 SRs/MAs ere included in this overview. The study selection process is summarized in Figure 2.

### Characteristics of included systematic reviews/meta-analyses

Table 1 summarizes the general characteristics of the 42 SRs/MAs included (year of publication and authors, number of included studies, total sample size, outcome indicators and prevalence, quality assessment tools, and journal of publication). A total of 40 of the SRs/MAs were published in English and the remaining two were in Chinese. Of these 42 SRs/MAs, 19 were reported by HCWs, 10 by CSs, four by CPs and nine by the GPs. Among the 42 SRs/MAs, 36 used quality assessment tools, of which JBI was the most common assessment tool, with five SRs/MAs using AHRQ, followed by one using AMSTAR-2, 12 using JBI, three using MMAT, one using MNOS, eight using NOS, one using ROBINS I, and four using STROBE. The outcome indicators observed in our study were mainly anxiety, depression and insomnia, and not all SRs/MAs included contained these three indicators. There were 34 SRs/MAs on anxiety, with a total of 6,028,108 samples reporting a comorbidity of anxiety, 31 on depression, with a total of 6,200,110 samples reporting a comorbidity of depression, and 18 on insomnia, with a total of 492,314 samples reporting a comorbidity of insomnia.

**TABLE 1:** Characteristics of included systematic reviews (*n* = 42).

Study ID	Study population	Sample size	Included research	Outcomes			Quality assessment tools
				Anxiety	Depression	Insomnia	
Pappa et al., 2020	HCW	33,062	13	23%	23%	34%	NOS
Al Maqbali et al., 2021	HCW	93,112	93	37%	35%	43%	NOS
Varghese et al., 2021	HCW	17,100	27	33%	32%	38%	NA
Ślusarska et al., 2022	HCW	44,165	23	29%	22%		AHRQ
Salari et al., 2020b	HCW	22,380	19	26%	24%		STROBE
Li et al., 2021	HCW	97,333	65	22%	22%		NA
Marvaldi et al., 2021	HCW	101,017	70	30%	31%	44%	AHRQ
Saragih et al., 2021	HCW	53,784	38	40%	37%		JBI
Batra et al., 2020	HCW	79,437	65	34%	32%	28%	NIH
Santabárbara et al., 2021a	HCW	57,430	71	25%			JBI
Norhayati et al., 2021	HCW	149,925	78	35%	35%	38%	JBI
Sahebi et al., 2021	HCW	187,506	96			36%	AMSTAR-2
Salari et al., 2020c	HCW	5,868	7			35%	STROBE
Johns et al., 2022	HCW	21,112	55	20%	26%		JBI
Hu et al., 2022	HCW	98,535	71	35%	38%	45%	NOS
Jahrami et al., 2021	HCW	54,231	44			36%	JBI
Serrano-Ripoll et al., 2020	HCW	119,189	117	30%	24%		ROBINS I
Serrano-Ripoll et al., 2021	HCW	13,486	13			38%	NA
Liu et al., 2021a	HCW	38,372	21	44%	31%		AHRQ
Mulyadi et al., 2021	CS	13,247	17	32%	52%	27%	JBI
Chang et al., 2021	CS	144,010	16	31%	34%		NOS
Deng et al., 2021a	CS	1,441,828	89	32%	34%	33%	NOS
Liyanage et al., 2021	CS	1,088,619	36	41%			JBI
Luo et al., 2021	CS	1,292,811	84		26%		JBI
Santabárbara et al., 2021d	CS	6,141	15	35%			JBI
Santabárbara et al., 2021c	CS	4,147	13		37%		JBI
Zhang et al., 2021	CS	203,678	31	24%	22%		AHRQ
Lasheras et al., 2020	CS	11,710	8	28%			JBI
Zhai et al., 2022	CS	128,536	38	24%			NA
Deng et al., 2021b	CP	5,153	31	47%	45%	34%	NOS
Cénat et al., 2021	CP	189,159	68	15%	16%		JBI
Liu et al., 2021	CP	4,381	22	38%	38%	48%	MNOS
Lao et al., 2020	CP	2,206	8		44%		NA
Liu et al., 2021b	GP	146,139	71	33%	28%	30%	NOS
Chen et al., 2022	GP	1,704,072	341	27%	30%		MMAT
Balakrishnan et al., 2022	GP	201,953	82		34%		STROBE
Zhang et al., 2022	GP	196,950	62	35%	35%	35%	MMAT
Pappa et al., 2022	GP	20,352	25	22%	16%		MMAT
Salari et al., 2020a	GP	44,531	17	32%	34%		STROBE
Santabárbara et al., 2021b	GP	56,679	43	25%			JBI
Nochaiwong et al., 2021	GP	284,813	32	27%	28%	28%	NA
Necho et al., 2021	GP	63,439	16	38%	34%		NOS

HCW, healthcare worker; CS, College student; CP, COVID-19 patient; GP, general population; AHRQ, agency for healthcare research and quality; AMSTAR-2, A measurement tool to assess systematic reviews; JBI, Joanna Briggs institute tool; MMAT, mixed methods appraisal tool; STROBE, strengthening the reporting of observational studies in epidemiology; NOS, Newcastle-Ottawa Scale; NIH, National Institutes of Health quality assessment tool; ROSBIN I risk of bias in non-randomized studies of interventions; MNOS modified form of the Newcastle-Ottawa scale; NA, not assessment.

### The methodological quality of included systematic reviews/meta-analyses

**FIGURE 3:**
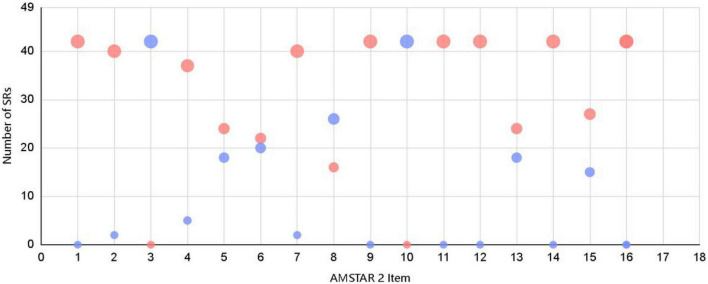
The bubble plot of quality assessment results.

The methodological quality of the 42 SRs/MAs included in the overview was evaluated using the AMSTAR 2 tool and the results are presented in the Table 2. the median AMSTAR 2 score was 11. of these 42 SRs/MAs, a total of 13 SRs/MAs met and partially met ≥70% of the entries, indicating high quality. For each entry of the AMSTAR 2 tool, the satisfaction was: item 1 (42/42, 100%), item 2 (40/42, 95.2%), item 3 (0/42, 0%), item 4 (37/42, 88.1%), item 5 (24/42, 57.1%), item 6 (22/42, 52.4%), item 7 (40/42, 95.2%), item 8 (16/42, 38.1%), item 9 (42/42, 100%), item 10 (0/42, 0%), item 11 (42/42, 100%), item 12 (42/42, 100%), item 13 (24/42, 57.1%), item 14 (42/42, 100%), item 15 (27/42, 64.3%), item 16 (42/42, 100%). The methodological quality of each SRs/MAs varied greatly and had some limitations. Among them, items 1, 9, 11, 12, 14, and 16 could be satisfied in each report. However, items 3 and 10 were poorly satisfied. The rest of the items were satisfied to vary degrees per SRs/MAs. Thirteen SRs/MAs were rated as “medium” for methodological quality, 13 were rated as “low,” and 16 were rated as “very low.” Subsequently, we visualized the quality assessment results using bubble plot (see Figure 3).

**TABLE 2:** A measurement tool to assess systematic reviews (AMSTAR 2) score for methodological quality of included systematic reviews.

Included studies	Item 1	Item 2	Item 3	Item 4	Item 5	Item 6	Item 7	Item 8	Item 9	Item 10	Item 11	Item 12	Item 13	Item 14	Item 15	Item 16	Total score
Pappa et al., 2020	Y	Y	N	Y	Y	Y	Y	N	Y	N	Y	Y	Y	Y	N	Y	12
Al Maqbali et al., 2021	Y	Y	N	Y	Y	Y	Y	N	Y	N	Y	Y	Y	Y	N	Y	12
Varghese et al., 2021	Y	Y	N	Y	N	N	Y	N	Y	N	Y	Y	N	Y	N	Y	9
Ślusarska et al., 2022	Y	Y	N	Y	N	Y	Y	Y	Y	N	Y	Y	N	Y	N	Y	10
Salari et al., 2020b	Y	Y	N	N	Y	N	Y	N	Y	N	Y	Y	N	Y	Y	Y	9
Li et al., 2021	Y	Y	N	Y	N	N	Y	N	Y	N	Y	Y	Y	Y	Y	Y	10
Marvaldi et al., 2021	Y	Y	N	Y	N	N	Y	N	Y	N	Y	Y	N	Y	Y	Y	9
Saragih et al., 2021	Y	Y	N	Y	N	N	Y	Y	Y	N	Y	Y	N	Y	N	Y	9
Batra et al., 2020	Y	Y	N	Y	Y	Y	Y	Y	Y	N	Y	Y	N	Y	Y	Y	12
Santabárbara et al., 2021a	Y	Y	N	Y	Y	N	Y	Y	Y	N	Y	Y	N	Y	N	Y	10
Norhayati et al., 2021	Y	Y	N	Y	Y	Y	Y	Y	Y	N	Y	Y	N	Y	N	Y	11
Sahebi et al., 2021	Y	Y	N	Y	Y	N	Y	N	Y	N	Y	Y	N	Y	N	Y	9
Salari et al., 2020c	Y	Y	N	Y	Y	N	Y	Y	Y	N	Y	Y	Y	Y	Y	Y	12
Johns et al., 2022	Y	Y	N	Y	Y	Y	Y	Y	Y	N	Y	Y	Y	Y	Y	Y	13
Hu et al., 2022	Y	Y	N	Y	Y	N	Y	N	Y	N	Y	Y	Y	Y	N	Y	10
Jahrami et al., 2021	Y	Y	N	Y	Y	Y	Y	Y	Y	N	Y	Y	Y	Y	Y	Y	13
Serrano-Ripoll et al., 2020	Y	Y	N	Y	N	Y	Y	Y	Y	N	Y	Y	N	Y	Y	Y	11
Serrano-Ripoll et al., 2021	Y	Y	N	Y	Y	N	Y	Y	Y	N	Y	Y	N	Y	Y	Y	11
Liu et al., 2021a	Y	Y	N	Y	N	Y	Y	N	Y	N	Y	Y	Y	Y	Y	Y	12
Mulyadi et al., 2021	Y	Y	N	Y	Y	Y	Y	N	Y	N	Y	Y	N	Y	Y	Y	12
Chang et al., 2021	Y	Y	N	N	N	Y	Y	N	Y	N	Y	Y	Y	Y	Y	Y	10
Deng et al., 2021a	Y	Y	N	Y	Y	N	Y	N	Y	N	Y	Y	Y	Y	Y	Y	11
Liyanage et al., 2021	Y	Y	N	Y	Y	N	Y	N	Y	N	Y	Y	Y	Y	N	Y	10
Luo et al., 2021	Y	Y	N	Y	N	N	Y	N	Y	N	Y	Y	N	Y	Y	Y	9
Santabárbara et al., 2021d	Y	Y	N	Y	Y	N	Y	N	Y	N	Y	Y	Y	Y	Y	Y	11
Santabárbara et al., 2021d	Y	Y	N	Y	Y	N	Y	N	Y	N	Y	Y	Y	Y	Y	Y	11
Zhang et al., 2021	Y	Y	N	Y	Y	Y	Y	Y	Y	N	Y	Y	N	Y	N	Y	11
Lasheras et al., 2020	Y	Y	N	Y	Y	Y	Y	Y	Y	N	Y	Y	Y	Y	Y	Y	13
Zhai et al., 2022	Y	N	N	Y	N	Y	N	N	Y	N	Y	Y	Y	Y	Y	Y	10
Deng et al., 2021b	Y	Y	N	Y	Y	Y	Y	N	Y	N	Y	Y	Y	Y	Y	Y	12
Cénat et al., 2021	Y	Y	N	N	N	N	Y	N	Y	N	Y	Y	Y	Y	Y	Y	9
Liu et al., 2021	Y	Y	N	Y	N	N	Y	N	Y	N	Y	Y	N	Y	N	Y	8
Lao et al., 2020	Y	Y	N	Y	N	N	Y	N	Y	N	Y	Y	N	Y	N	Y	8
Liu et al., 2021b	Y	N	N	Y	N	Y	N	N	Y	N	Y	Y	Y	Y	Y	Y	10
Chen et al., 2022	Y	Y	N	Y	N	N	Y	N	Y	N	Y	Y	Y	Y	Y	Y	11
Balakrishnan et al., 2022	Y	Y	N	N	N	Y	Y	N	Y	N	Y	Y	Y	Y	Y	Y	11
Zhang et al., 2022	Y	Y	N	N	Y	Y	Y	Y	Y	N	Y	Y	N	Y	N	Y	11
Pappa et al., 2022	Y	Y	N	Y	N	Y	Y	Y	Y	N	Y	Y	Y	Y	Y	Y	13
Salari et al., 2020a	Y	Y	N	Y	Y	N	Y	N	Y	N	Y	Y	N	Y	N	Y	10
Santabárbara et al., 2021b	Y	Y	N	Y	Y	Y	Y	Y	Y	N	Y	Y	Y	Y	Y	Y	14
Nochaiwong et al., 2021	Y	Y	N	Y	Y	Y	Y	Y	Y	N	Y	Y	Y	Y	Y	Y	14
Necho et al., 2021	Y	Y	N	Y	N	Y	Y	N	Y	N	Y	Y	Y	Y	Y	Y	12

Y, when the criterion is explicitly met; N, when the criterion is explicitly not met.

Item 1 Did the research questions and inclusion criteria for the review include the components of PICO?

Item 2 Did the report of the review contain an explicit statement that the review methods were established prior to the conduct of the review and did the report justify any significant deviations from the protocol?

Item 3 Did the review authors explain their selection of the study designs for inclusion in the review?

Item 4 Did the review authors use a comprehensive literature search strategy?

Item 5 Did the review authors perform study selection in duplicate?

Item 6 Did the review authors perform data extraction in duplicate?

Item 7 Did the review authors provide a list of excluded studies and justify the exclusions?

Item 8 Did the review authors describe the included studies in adequate detail?

Item 9 Did the review authors use a satisfactory technique for assessing the risk of bias (RoB) in individual studies that were included in the review?

Item 10 Did the review authors report on the sources of funding for the studies included in the review?

Item 11 If meta-analysis was performed, did the review authors use appropriate methods for statistical combination of results?

Item 12 If meta-analysis was performed, did the review authors assess the potential impact of RoB in individual studies on the results of the meta-analysis or other evidence synthesis?

Item 13 Did the review authors account for RoB in primary studies when interpreting/discussing the results of the review?

Item 14 Did the review authors provide a satisfactory explanation for, and discussion of, any heterogeneity observed in the results of the review?

Item 15 If they performed quantitative synthesis did the review authors carry out an adequate investigation of publication bias (small study bias) and discuss its likely impact on the results of the review?

Item 16 Did the review authors report any potential sources of conflict of interest, including any funding they received for conducting the review?

### Subgroup analysis of psychological disorder symptoms

#### Anxiety

**FIGURE 4:**
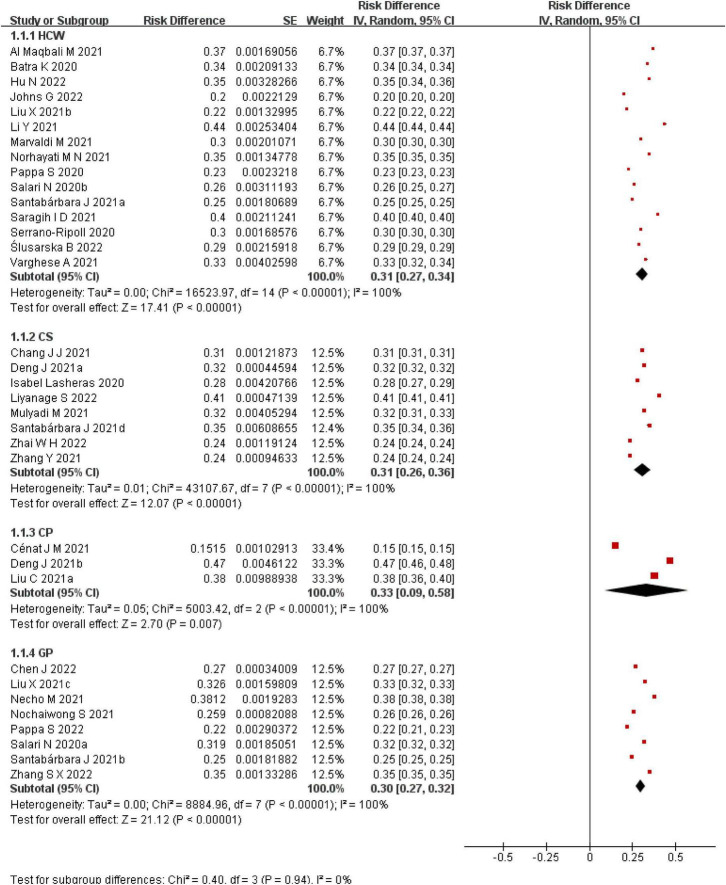
A meta-analysis of the prevalence of anxiety.

A total of 34 SRs/MAs on the prevalence of anxiety are available. The prevalence of anxiety in different populations during the COVID-19 pandemic is analyzed in this overview (see Figure 4). Significance tests of the results of the group comparisons revealed no statistically significant differences (*p* = 0.94). In the HCWs population, the results of 15 SRs/MAs were summarized and the interval of anxiety prevalence was found to be 20–44%, among which Batra et al. (2020), Liu et al. (2021a) and Saragih et al. (2021) reported significantly higher prevalence of anxiety in HCWs than others. Liu et al. (2021a) study subjects were all from the sentinel hospital (a hospital that concentrates mainly on treating CPs), and the work pressure of HCWs in this hospital was higher, resulting in a higher prevalence of anxiety. In the CSs group, a summary of eight reports found a prevalence range of 24–41%. In the CPs cohort, three reports had a prevalence range of 15–47%, and the results of these three reports differed significantly. The meta-analysis of Deng et al. (2021b) had a high AMSTAR 2 score and high confidence in the outcome. the reports of Cénat et al. (2021) and Liu et al. (2021) were “very low” quality studies in terms of methodological quality assessment and both reports had problems with literature search, data extraction and publication bias, resulting in low credibility of the final results. In the GPs population, a summary of the results of eight SRs/MAs found a prevalence range of 22–38%.

#### Depression

**FIGURE 5:**
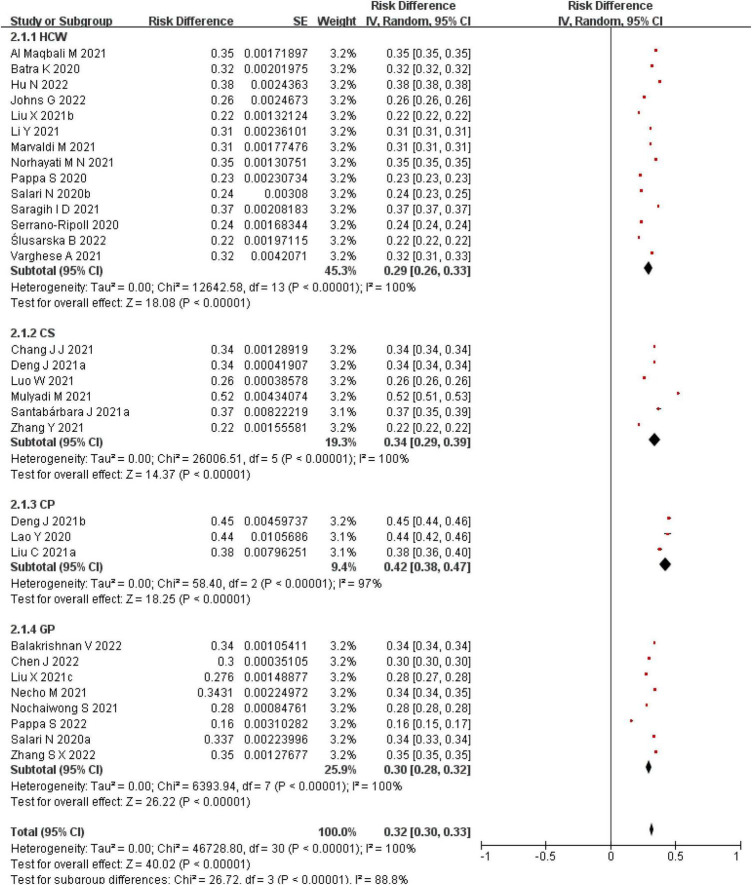
A meta-analysis of the prevalence of depression.

A total of 30 SRs/MAs on the prevalence of depression are presented in this overview to analyze the prevalence of depression in different populations during the COVID-19 pandemic (see Figure 5). The results of the comparison between groups were statistically significantly different (*p* < 0.05). In the HCWs group, the results of 14 SRs/MAs were summarized and the range of depression prevalence was found to be 22–38%. In the CSs group, summarizing the results of six SRs/MAs found a depression prevalence interval of 22–52%. The results of the meta-analysis by Mulyadi et al. (2021) differed from the others, but the study had a better qualitative assessment and higher confidence. We analyzed the reason for this and found that unlike others the study was conducted with all nursing medical students. The authors of this article explain that nursing students had a high prevalence of depression before the COVID-19 pandemic, probably due to educational and family factors, and that there was a significant increase in prevalence during the pandemic. The intervals of depression prevalence were 38–45% and 16–35% in the CPs and GPs groups, respectively.

#### Insomnia

**FIGURE 6:**
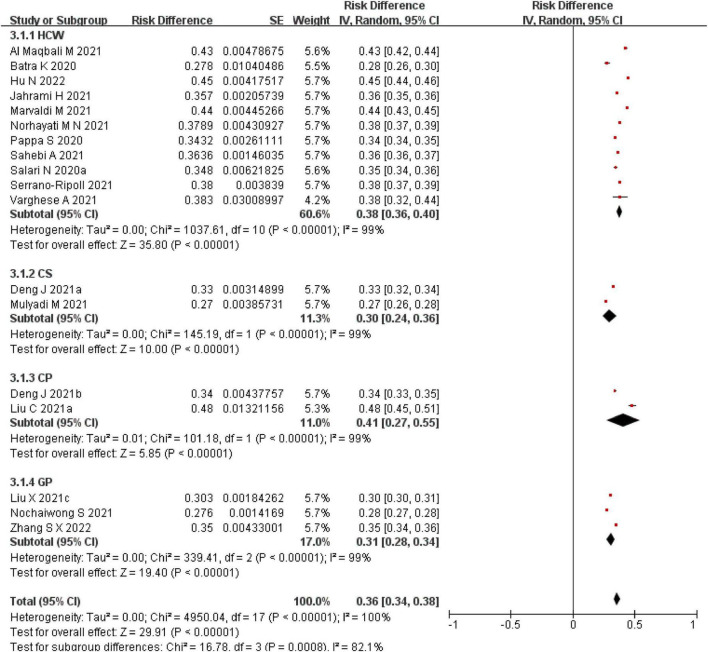
A meta-analysis of the prevalence of insomnia.

There were 18 SRs/MAs on the prevalence of insomnia in different groups (HCWs, CSs, CPs, GPs). The results of the comparison between groups were statistically significantly different (*p* < 0.05). Summary results found that the intervals of insomnia prevalence were 28–45%, 27–33%, 34–48%, 28–35%, respectively (see Figure 6).

## Discussion

### Statement of main findings

The prevalence of psychological disorders in various populations increased significantly during the COVID-19 pandemic (Pfefferbaum and North, 2020), and the purpose of this review was to assess the methodological quality of the SRs/MAs and to provide an description of the occurrence of anxiety, depression, and insomnia in different populations. We performed a meta-analysis of the prevalence of anxiety, depression and insomnia in the target study population. We found that the prevalence of anxiety in the different subgroups were: HCWs (20–44%), CSs (24–41%), CPs (15–47%), and GPs (22–38%), but there were no statistically significant differences between this subgroup comparison (*p* = 0.94). The prevalence of depression were: HCWs (22–38%), CSs (22–52%), CPs (38–45%), and GPs (16–35%). Our results show that the prevalence of depression is higher in CPs and CSs than in the rest of the population, with a statistically significant difference in the comparison between this subgroup (*p* < 0.05). We need to pay extra attention to the mental health problems of CSs. Chen et al. argued that adolescents are immature and belong to a vulnerable group, and that CSs are prone to depressive symptoms and subsequent development of depression after the outbreak of the new crown pandemic, when they spend long periods of time taking courses online and doing related activities indoors, and this change in lifestyle and the threat of possible infection (Chen F. et al., 2020). In terms of insomnia-related prevalence, the results for the different subgroups were: HCWs (28–45%), CSs (27–33%), CPs (34–48%), and GPs (28–35%), with statistically significant differences (*p* < 0.05). We then tested the heterogeneity of the included studies, first using a fixed-effects model for heterogeneity, and the results showed (*I*^2^ > 50%) a large heterogeneity among studies, and then we changed to a random-effects model, where the same heterogeneity existed. This may be due to the fact that the included SRs/MAs were from studies conducted by scholars in various countries around the world, and since the pandemic was global in nature, the raw data for the included SRs/MAs were from different countries and regions. The severity of the epidemic and prevention and control measures vary from country to country, resulting in differences in the prevalence of mental illness in the population, so there is a high degree of heterogeneity among studies. However, the purpose of our study was to summarize the prevalence of anxiety, depression, and insomnia in key populations worldwide during the COVID-19 pandemic, so the presence of heterogeneity was unavoidable for our study purpose and did not substantially affect our study purpose. For the results of inter-group comparison of insomnia prevalence, we found that patients with COVID-19 were the population with the highest occurrence of psychological disorders, and sleep disorders continued to be the most common psychological disorder in patients with COVID-19, possibly due to the core symptoms of COVID-19 including cough, fever and dyspnea, all of which are associated with sleep problems (Ferrando et al., 2016; Huang et al., 2020). It has been suggested that the higher risk of sleep problems in CPs may also be attributable to physical pain and side effects of medications used to treat the virus (Shi et al., 2020). The overall analysis showed that the prevalence of insomnia was the highest, and a previous meta-analysis showed that the bulk of sleep disorders in the GP was only 15% during non-COVID-19 pandemics (Cao et al., 2017). It has been suggested that the reason why insomnia is more common is because of the potential bidirectional relationship between sleep and psychiatric co-morbidities, especially when more co-morbidities are present (e.g., anxiety and depression), which suggests that sleep specialists treating this suggests that sleep specialists should consider psychiatric co-morbidities when treating sleep problems, and vice versa (Jahrami et al., 2021).

Our results indicate that insomnia-related symptoms were more common in different populations during the new crown epidemic relative to anxiety and depression. A previous meta-analysis showed that the bulk of sleep disorders in the GP was only 15% during non-COVID-19 pandemics (Cao et al., 2017). It has been suggested that the reason why insomnia is more common is because of the potential bidirectional relationship between sleep and psychiatric co-morbidities, especially when more co-morbidities are present (e.g., anxiety and depression), which suggests that sleep specialists treating this suggests that sleep specialists should consider psychiatric co-morbidities when treating sleep problems, and vice versa (Jahrami et al., 2021). In addition, due to the lack of knowledge about the COVID-19 and the huge lethality caused by the lack of effective treatment measures at the beginning of the pandemic, patients with the COVID-19 are filled with internal panic and fear for their lives (Gyasi, 2020), the lack of contact with the outside world during isolation or hospitalization, which makes them more likely to suffer from loneliness, anxiety and depression, and even suicidal thoughts (Kawohl and Nordt, 2020; Luchetti et al., 2020; Pappa et al., 2020; Varghese et al., 2021). The prevalence of psychological disorders among HCWs is also higher, probably due to the huge workload during the pandemic, the enormous psychological pressure on HCWs, coupled with the fear of being infected by contact with CPs, as well as the need for a series of measures such as isolation when leaving the hospital, creating a very obvious psychological barrier for HCWs (Pappa et al., 2020; Al Maqbali et al., 2021; Marvaldi et al., 2021; Saragih et al., 2021; Varghese et al., 2021).

### Methodological quality of systematic reviews

In terms of methodological quality, of the 42 SRs/MAs included, 16 were of very low-quality, the rest were of low-quality and moderate-quality, and there was no high-quality SRs/MAs. Problems were more pronounced in three areas: (1) The inclusion criteria for the type of study were not specified, only the inclusion of cross-sectional studies was described, and the reasons for the inclusion of study types were not explained; and (2) All studies only described the reasons for exclusion of literature without providing a detailed list of excluded literature; Also for the very low quality 16 SRs/MAs, the problems were mainly related to the impact on the risk of bias not being explained in the discussion. These results suggest that the current SRs/MAs exploring the incidence of anxiety, depression, and insomnia in different populations during the pandemic generally follow the reporting norms. However, the methodological quality needs to be improved, and researchers still lack attention in explaining the type of study design, providing a list of excluded literature, and the reasons and sources of funding.

### Strengths and limitations

This overview is the first study to assess the quality of evidence on the incidence of anxiety, depression, and insomnia in different populations during the COVID-19 pandemic using AMSTAR 2 tools. However, our study has some limitations. First, in this review, we included only SRs/MAs, while primary studies (e.g., cohort studies, observational studies, and case-control studies) were not reviewed. Second, only Chinese and English literature were included in this study, relevant gray literature was not obtained, and the search results may be subject to publication bias. Third, the subjectivity of the evaluators in evaluating the literature may lead to bias and thus affect the evaluation results.

## Conclusion

The evidence summarized in this paper suggests that the methodological quality of SRs/MAs is not high. Therefore, they should be improved using the AMSTAR 2 tool to provide effective evidence-based medicine for targeted psychological interventions, psychological counseling services, and adequate social support help to alleviate psychological disorders due to pandemic factors.

## Data availability statement

The original contributions presented in this study are included in the article/Supplementary material, further inquiries can be directed to the corresponding author.

## Author contributions

QZ and SS designed the study. QZ and YT searched the literature and performed screening. CJ and PL collected relevant data. QZ analyzed the data and drafted the manuscript. JT and SS revised and approved the final version of the manuscript. All authors have read and approved the submitted version.
