# Supplementary material for: Prevalence of anxiety, depressive and insomnia symptoms among the different groups of people during COVID-19 pandemic: An overview of systematic reviews and meta-analyses
Source: Front Psychol. 2022 Nov 16;13:1024668. doi: 10.3389/fpsyg.2022.1024668 (PMC9709262; doi:10.3389/fpsyg.2022.1024668)
Supplement: Supplementary file 1 [file Table_1.DOCX]

Supplementary Material

# Supplementary Tables

**Supplement Table 1** Definition of quality levels for AMSTAR 2 scores

| Grade |  | **Definition** |  |
| --- | --- | --- | --- |
| High |  | None or only 1 non-vital entry not met: for the research question, the systematic evaluation provided an accurate and comprehensive summary based on the results of the accessible studies. | |
|  |  |  |  |
| Medium |  | More than 1 non-vital entry was not met*: based on the results of accessible studies, the systematic evaluation may have provided an accurate summary. | |
|  |  |  |  |
| Low |  | 1 vital entry not met and with or without non-vital entry not met: based on the results of the available studies, the systematic evaluation may not provide an accurate and comprehensive summary. | |
|  |  |  |  |
| Very low |  | More than 1 vital entry not met, with or without non-vital entry not met: based on the results of the accessible studies, the systemic evaluation can't provide an accurate and comprehensive summary. | |
|  |  |  |  |

*: When multiple non-vital entries are not met, it reduces confidence in the system's evaluation results and can be downgraded from medium to low.
